# Perception of nonrigid structures from motion using tracking image gradient vectors

**DOI:** 10.3389/fpsyg.2025.1586648

**Published:** 2025-08-26

**Authors:** Hiroshige Takeichi, Wataru Suzuki, Wakayo Yamashita, Atsushi Hiyama

**Affiliations:** ^1^Open Systems Information Science Special Team, Predictive Medicine Special Project (PMSP), RIKEN Center for Integrative Medical Sciences (IMS), RIKEN, Yokohama, Kanagawa, Japan; ^2^Computational Engineering Applications Unit, Head Office for Information Systems and Cybersecurity (ISC), RIKEN, Wako, Saitama, Japan; ^3^Machine Intelligence for Medical Engineering Team, Center for Advanced Intelligence Project, RIKEN, Chuo-ku, Tokyo, Japan; ^4^Department of Information Science and Biomedical Engineering, Graduate School of Science and Engineering, Kagoshima University, Kagoshima, Japan; ^5^Research Center for Advanced Science and Technology, The University of Tokyo, Meguro-ku, Tokyo, Japan; ^6^Graduate School of Social Data Science, Hitotsubashi University, Kunitachi, Tokyo, Japan

**Keywords:** optical flow, gradient-based feature tracking, motion vector field, perceptual augmentation, nonrigid structure from motion, middle-level vision, perception science

## Abstract

**Introduction:**

A subset of the true optical flow can be extracted by constructing a vector field that represents image gradients and then tracking vectors in this vector field. This pseudo-flow (p-flow) subset effectively visualizes nonrigid motion and leads to the perception of nonrigid structure from motion. In this study, we investigate whether the human sensory system can extract information about the physical properties of inanimate fluid, especially viscosity, from the p-flow.

**Methods:**

Computer-generated movies of flowing liquid were constructed using the p-flow algorithm and the Lucas–Kanade method. The movies featured liquids of different viscosities in the form of point-light displays. The viscosity of the fluid in various subsets of these movies was then estimated by 312 participants.

**Results:**

The error, i.e., difference between expected and actual ratings showed smaller variability across repeated trials and the mean response time was significantly shorter when using the p-flow than with the conventional Lucas–Kanade method.

**Discussion:**

Our results suggest that the p-flow enables a more reliable viscosity rating, which could be related to the local constraint used in the algorithm.

## 1 Introduction

Visual motion provides a variety of information types that are collectively referred to as interpretation, such as depth in space, segmentation and shape of objects, and self-locomotion (Nishida et al., 2018). The human sensory system can extract global information pertaining to visual motion by integrating multiple local movements in spatially restricted portions of the visual field (Hedges et al., 2011), e.g., in the form of a point light display. This is because the global motion of an object or scene with a three-dimensional (3D) physical constraint imposes spatiotemporally structured motion on individual points, and the sensory system can reconstruct the global motion constraint from the point light display (Ullman, 1984; Grzywacz and Hildreth, 1987; Jackson and Blake, 2010; Thurman and Lu, 2013).

Psychophysical and neurophysiological experiments have shown that the human sensory system can extract various information from point light displays, including biological motion (Johansson, 1973, 1976; Cutting and Kozlowski, 1977), the structure of objects (Treue et al., 1991), the rotation direction of objects (Pollick et al., 1994; Grunewald et al., 2002), and depth in space (Treue et al., 1995). Biological motion not only provides information about the state of the agent, i.e., walking, running, and direction of motion, but also enables identification of the gender (Cutting et al., 1978; Mather and Murdoch, 1994; Pollick et al., 2005), age, and emotion of the agent (Dittrich et al., 1996). Perception of biological motion requires knowledge of the body skeleton and kinematics (Jackson and Blake, 2010; Thurman and Lu, 2013).

By contrast, Kawabe et al. (2015) showed that the viscosity of a liquid can be accurately estimated from motion using evenly spaced Gaussian noise that dynamically changes following the motion vector field. In their study, the motion vector field was calculated using the Lukas–Kanade (LK) method (Lucas and Kanade, 1981). The LK method is a gradient-based approach for optical flow extraction that solves partial differential equations under the constant brightness assumption (Tu et al., 2019). This constraint is introduced because the algorithm of motion detection depends on the measurement of motion energy or spatiotemporal gradients.

In general, gradient-based approaches such as the LK method are not suitable for visualizing the motion extracted by tracking certain features. If motion detection depends on the tracking of image features, it is classified as a feature-based approach, rather than a gradient-based approach. Point light displays of a solid animate or inanimate object can be created by placing point lights at positions that can be tracked, such as the head, arms, and legs in the case of biological motion. However, it is generally difficult to reproduce the motion of fluids using point light displays because there are no such apparent landmarks, unless a point cloud with a physical simulation is created using computer graphics.

We have previously developed a motion extraction algorithm with a feature-based approach that is also consistent with gradient-based techniques (Suzuki et al., 2017, 2019, 2020). The motion information extracted using this algorithm is referred to as the pseudo-flow (p-flow) because it is a subset of, and therefore not identical to, the exact ground-truth optical flow. The p-flow algorithm does not track points by matching static features, but instead constructs a vector field of the image gradient, i.e., the spatiotemporal derivative of an image, and then matches (tracks) vectors in this vector field. Because the p-flow algorithm is consistent with the two approaches, it produces the point light displays of fluid motion using the advantage of the feature-based approach while it is expected that the viscosity of the fluid is successfully estimated because of the characteristics of the gradient-based approach as is indicated by Kawabe et al. (2015).

Gradient-based motion processing is related to the first-order visual motion system (Lu and Sperling, 2001) in that both take image gradients as their input and both are based on opponent directional selectivity, and the feature-based motion processing to the third-order visual motion system in that both track salient features. Because motion detection by gradient-based processing depends on opponency, i.e., antagonism between a pair of detectors with opposing directional selectivity (van Santen and Sperling, 1985), it is inherently insensitive to the motion signal along the orthogonal orientation. This is called the aperture problem and is generally assumed to be resolved by integrating local motion signal from the same source but along different orientation axes at a later stage (Adelson and Movshon, 1982), when suitable groupings are made based on some perceptual organization. In contrast, motion detection by feature-based processing depends on the choice of the features to be tracked. Thus, it does not suffer from the aperture problem when the features are unique and therefore their movements can be measured unambiguously. In a sense, gradient-based processing suffers from the aperture problem and therefore it solves the problem by subsequent motion integration, whereas feature-based processing does not suffer from the problem because it resolves the problem by prior selection of unambiguous features.

The insight of the p-flow algorithm is that the spatiotemporal gradient vector itself constrains the range of possible motion interpretation if it is matched and tracked between several time points. Whereas p-flow integrates gradient-based and feature-based approaches, we do not propose our version of one-system theory as an alternative to the three systems theory. The present study does not concern what types of or how such motion signals are detected, but rather concerns perceptual organization of such motion signals, namely, integration of temporal and spatial changes. For such integration, vectors of spatiotemporal image gradients are matched and tracked as features in the p-flow algorithm.

The matched vectors may be visualized by representing them with moving dots in a point light display. Psychophysical studies showed that the p-flow algorithm successfully extracts the vection-inducing component of animated films (Suzuki et al., 2019), as well as the motion of nonbiological fluids in informal observations (Suzuki et al., 2020), which suggest effective extraction of perceptually organized motion signal components by the p-flow. In this study, we investigate how accurately and reliably viscosity can be perceived, as a benchmark for processing nonrigid structure from motion, in movies of a flowing liquid through movements in point-light displays reconstructed by the p-flow, and compare the results with those given by the LK method.

## 2 Methods

### 2.1 Ethics statement

The data collection and analysis were conducted in accordance with the Declaration of Helsinki. The protocol of the human experiment conducted as part of this study was approved prior to initiation by the Wako 3rd Ethics Committee of RIKEN (Wako3 2021-13).

### 2.2 Participants

Participants were recruited through a market research agent (Cross Marketing Inc., Tokyo, Japan) such that they had a uniform distribution of sex and age. All participants reported that they had a visual acuity of 0.3 or greater for each eye monocularly and 0.7 or greater binocularly, with correction if needed, and that they used one of several specified types of iPhone (Apple Inc. Cupertino, CA, USA), as described below.

Informed consent was obtained from each participant via the first page of the website used to conduct the experiment. The experimental tests appeared on subsequent pages. If more than 60 min elapsed after a participant had established a connection, the participant was considered to have withdrawn. The participants were only paid if they completed the experiment, as explained prior to participation and in accordance with the approval by the ethics committee.

Crowdsourcing was not used in this study because of concerns about participant demographics (Ware et al., 2019) and maintaining motivation (Sasaki and Yamada, 2019). Instead, recruitment was outsourced to a marketing research agent. This enabled the exclusive use of a specific type of device, i.e., an iPhone, as described below, for the experiment, thus effectively mitigating problems associated with variations in the display, such as the stimulus size (Pechey et al., 2015).

### 2.3 Stimulus and apparatus

Three sets of movies were created: a training set, a practice set, and a measurement set, all of which were adapted from the supplemental information of van Assen et al. (2018). The training set comprised movies simulating liquids with minimum and maximum viscosities of 0.0035938 and 7.7426 Pa·s, respectively, taken from Set 2 of their Experiment 2, in which there were eight scene variations (see their Figure 1A). The practice set comprised all movies in Set 2 of their Experiment 2 with seven levels of viscosity. The measurement set comprised 10 of the 32 movies in Set 1 of their Experiment 1 (see their Figure 2A). These 10 movies were selected by taking one of every three movies, evenly spaced in rank and with simulated viscosities ranging from 0.0020771 to 40.103 Pa·s. Thus, there were 16 training, 56 practice, and 10 measurement movies. For each participant, two movies were randomly sampled from the 16 training movies and three movies were randomly sampled from the 56 practice movies. All 10 measurement movies were used for all participants, but each participant was shown only one of two versions: the original movie and the p-flow movie.

**Figure 1:**
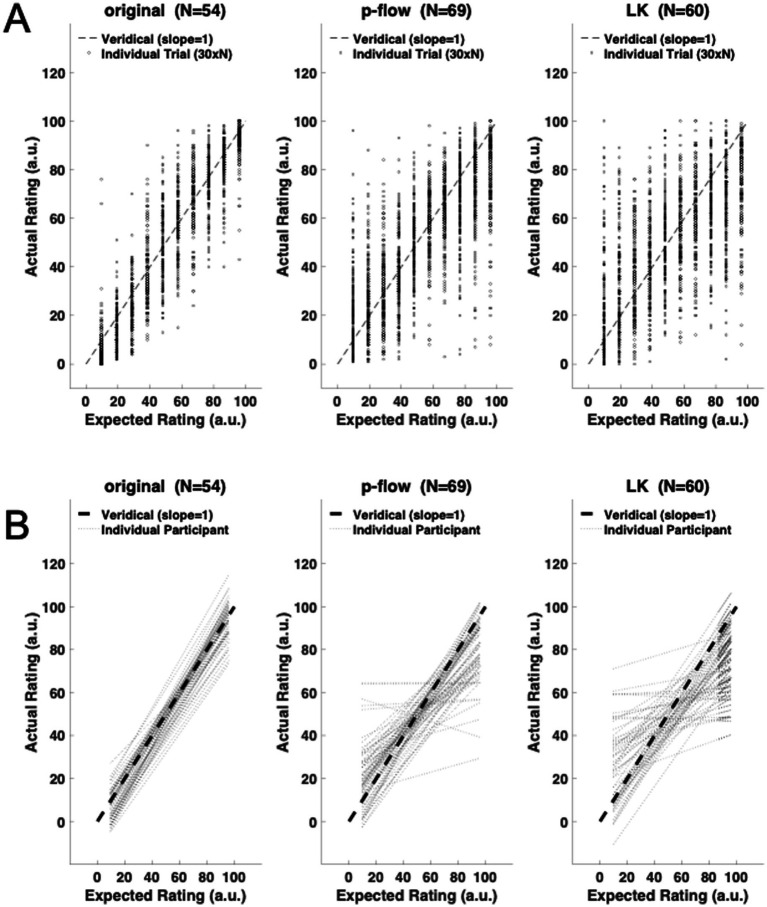
Change of median RMS error over trials for each algorithm. The rating performance changes greatly between the 1st and the 20th trials, but becomes more stable afterwards.

**Figure 2:**
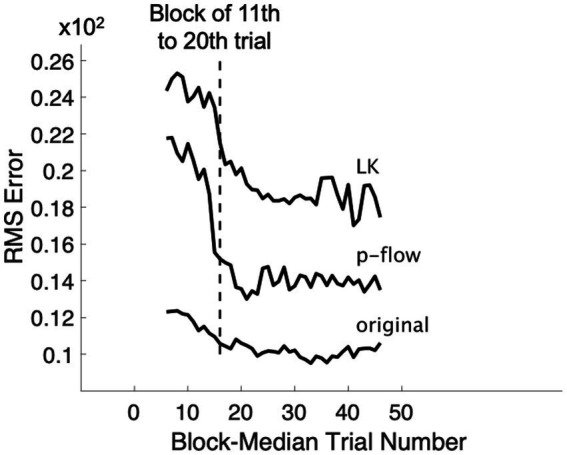
**(A)** Trial-wise scatter plot of the expected and actual ratings for each algorithm: the original movies, p-flow movies, and the optical flow movies made with the Lucas–Kanade (LK) method. Only data between the 21th and the 50th trials are shown (see Figure 1). The broken line represents the veridical response in which the expected and actual ratings are the same. **(B)** Participant-wise regression of the actual ratings for each of the movies. The slopes and the intercepts are mostly veridical, i.e., close to 1.0 and 0.0, respectively, but are less accurate in the p-flow than the original, and in the LK method than the p-flow for some participants. The broken line represents the veridical response in which the expected and actual ratings are the same.

The original movies (see Supplementary material Videos 1 through 5) were generated from those of van Assen et al. (2018) by decolorization and blurring using a box filter of 3 × 3 pixels. The p-flow movies (Supplementary material Videos 6 through 10) were composed of moving dots that showed the visual motion of the flowing liquid in the original movies, as extracted using the p-flow algorithm. The LK optical flow movies (Supplementary Material Videos 11 through 15) were similarly composed of moving dots that showed the visual motion, as extracted using the LK method implemented as a function in the Open Source Computer Vision Library (OpenCV). Each stimulus movie lasted 10 s (as described in the Method details/stimuli section of van Assen et al., 2018) and was played in this study as a YouTube (Google LLC, San Bruno, CA, USA) video embedded in a web page. The participants viewed the web page on their mobile device via their preferred browser, which was required to be either Safari (Apple Inc. Cupertino, CA, USA), Chrome (Google LLC, San Bruno, CA, USA), or Firefox (Mozilla, San Francisco, CA, USA). The device was required to be an iPhone 6, iPhone 6 plus, iPhone 6s, iPhone 6s plus, iPhone 7, iPhone 7 plus, iPhone 8, iPhone 8 plus, iPhone XR, iPhone 11, or the new model of iPhone SE sold in Japan after April 2020. These iPhone models were selected to limit the specifications of the display device: the screen size ranged from 4.7 to 6.1 in. (~12–15.5 cm), the resolution was either 326 or 401 pixels per inch, the contrast was 300 or 400, and the brightness was 500 or 625 cd/m^2^. Each participant was randomly assigned and asked to rate only one of the two versions in the measurement set. In contrast, the same set of movies was assigned to all participants for training and practice, regardless of the participant’s assigned measurement set.

In the instructions regarding viewing conditions, the participants were requested to be in isolation at home, to be stably connected to the internet, to turn off room lights, and to hold the device in landscape orientation to view the stimulus. In a typical situation, a 4-cm-wide (or high) stimulus on the screen should subtend approximately 9° at an observation distance of 25 cm, thus occupying the fovea and parafovea.

### 2.4 Procedure

The experiment was conducted online using a web page developed with a custom JavaScript code on a cloud server at https://pavlovia.org/ (Open Science Tools Ltd., Nottingham, UK) using jsPsych 6.0 (de Leeuw, 2015). The experiment could be conducted at any time of day, according to the participant’s preference. Each participant received video-based general instructions about the viewing conditions (described above) at the beginning of the experiment, after providing their informed consent.

Each participant performed a minimum of two and a maximum of six training trials, three practice trials, and 50 measurement trials. Written instructions for the task were presented before the first trials in the training and measurement phases. The first trial in each of these phases started when the participant tapped a button at the bottom of the instructions. The trial web page contained, from top to bottom, a stimulus movie, a response slider, and a response button. The stimulus movie was first automatically played silently in each trial. Following the movie, the participant was required to adjust the slider below the movie to indicate their viscosity rating, after which the participant was required to tap the button below the slider to proceed to the next trial. The button remained inactive, i.e., unresponsive, and was indicated as such by reduced contrast, during the first 10 s of the trial and was activated when the 10-s stimulus presentation had finished and the participant had moved the slider from its initial position of 50. The rating was made on a flexible 101-point scale from 0 to 100, as described below, with higher numbers corresponding to an impression of higher viscosity. The participant could play the stimulus movie several times at will by using a standard YouTube operation.

#### 2.4.1 Training trials

The two types of baseline movies were shown during the training phase to provide anchors for the rating. They were presented successively in separate trials, and the participant was required to associate the perceived viscosity with the normalized rating scale by following the instructions to adjust the slider to ratings of 10 and 80 for the low- and high-viscosity training movies, respectively. If the participant did not successfully adjust the slider to a value between 6 and 14 for 10 and between 76 and 84 for 80, the training trial on the same baseline stimulus was repeated up to two more times. After three repetitions for each baseline stimulus, the experiment proceeded to the next step, irrespective of the participant’s response.

#### 2.4.2 Practice trials

Three trials were conducted during the practice phase. The three stimulus movies were independently and randomly selected, with varying simulated viscosity and scene. Movies from different scenes were used to discourage the participant from relying on cues that were valid only in particular scenes and unrelated to viscosity. The participant was required to rate the perceived viscosity and to report the rated value by adjusting the slider for each stimulus on the scale defined by the two baseline anchor movies shown in the training phase. The expected rating, which was linearly proportional to the simulated viscosity on a logarithmic scale, was displayed as feedback after each response in the practice phase. The participant was required to tap a button to acknowledge the feedback and proceed to the next trial in the practice phase, or to proceed to the measurement phase at the end of the third trial.

#### 2.4.3 Measurement trials

There were 50 trials in the measurement phase. These began after the rating instructions had been repeated. The trials in the measurement phase were divided into five blocks, each containing 10 trials. Each of the 10 stimuli with 10 viscosity levels was presented once in pseudorandom order during the 10 trials per block. Different random sequences were used for different participants and in different blocks. As in the practice phase, the participant was required to rate the perceived viscosity and to report the rated value by adjusting the slider for each stimulus on the scale defined by the two baseline anchor movies in the training phase. No feedback was provided in the measurement phase. The recorded data were the rating indicated by the slider and the response time (RT), defined as the duration between the start of the movie and when the button was tapped to proceed to the next movie.

When an extreme value of 0 or 100 was recorded as the rating, the slider was expanded by 10 in the direction of the extremity in subsequent trials to avoid saturation of the rating scale. For example, if the participant gave a rating of 0, i.e., the minimum value, then the scale was expanded from [0, x] to [−10, x] in subsequent trials until the end of the experiment. Similarly, if the participant gave a rating of 100, i.e., the maximum value, then the scale was expanded from [x, 100] to [x, 110]. If the participant subsequently gave a rating of −10 on the expanded scale of [−10, x], then the scale was further expanded to [−20, x]. This process was repeated without any limit. The expansion of the scale was introduced because we were concerned about the participants’ not reporting exact ratings as they perceived but unnaturally rounding the ratings to fit to the scale that was bound at both ends. Because the scale expansion was only intended to mitigate such participants’ over-adaptation, data from participants with a value on an extended scale were not included in the actual analysis.

The trial was aborted if the RT reached 60 s. The experiment was discontinued if three trials were aborted or if 40 min or more had elapsed since the beginning of the experiment. These time limits were explained to the participants in advance.

### 2.5 Analysis

The mean and standard deviation of the rating error and mean RT were the three main dependent variables for individual participants. The rating error was calculated as the difference between the actual rating and the expected rating for each trial. The expected rating was proportional to the logarithm of the simulated viscosity. The mean and standard deviation of the rating error were subsequently calculated for each stimulus by averaging across repeated presentations for each participant. The mean RT was calculated similarly for each stimulus by averaging across repeated trials for each participant.

To evaluate the time-course in which the rating performance improved, the time series was first defined using a moving window of 10 trials. Because all 50 trials were included in the analysis, there were 41 moving windows. Each of the 41 moving windows was designed to comprise 10 trials in which each of the 10 stimuli was presented once. The initial window was composed of the first 10 trials, which covered all 10 stimuli. The subsequent windows covered all 10 stimuli as well, after the data were sorted for each of the 10 trial blocks to have the same order as the first 10 trials. Because initial inspection of the data revealed that the performance stabilized after 2 blocks, as Figure 1 shows through the average RMS error for each algorithm, the data from the first 2 blocks were excluded and those from the final 3 blocks were analyzed.

Finally, the following three check variables were evaluated: the number of aborted trials (i.e., those with an RT of longer than 60 s), the number of baseline trials that were necessary to respond as requested, and the RMS error for the practice trials. The reason for this evaluation was to check whether extraneous factors—such as participants’ seriousness, motivation, comprehension, or compliance with the experimental instructions—contributed to differences between algorithms.

### 2.6 Design and statistics

There were two independent variables: the type of algorithm used to generate the stimulus for the participant (original, p-flow or LK) and the expected response that corresponds to the log simulated viscosity for each stimulus. There were three dependent variables: the mean and standard deviation of the rating error, and the mean RT. Two-way analysis of variance (ANOVA) was performed for each of the three dependent variables and three check variables. The algorithm and the stimulus were the factors of two and 10 levels, respectively. *Post hoc* tests of multiple comparisons were performed by the Tukey method. Because six ANOVAs were performed (on the mean error and its standard deviation, mean RT, and three check variables), alpha was set to 0.00833 (= 0.05/6) by applying the Bonferroni correction for multiple comparisons. The data were analyzed using a custom-made code in MATLAB R2021a or later (MathWorks, Natick, MA, USA). ANOVA was performed using either MATLAB or Anova-kun 4.8.5 (Iseki, 2023) in R version 4.0.3 (R Core Team, 2023).

## 3 Results

Invitations were sent to 622 candidates, who participated in the experiment partially or fully. Of these, 312 volunteers (50.22%) completed the experiment (150 males and 162 females; 68, 95, 79, and 70 participants in the 20–29, 30–39, 40–49, and 50–59 age groups, respectively). The final responses to the first and second anchoring baseline stimuli were required to be in the ranges of [6, 14] and [76, 84], respectively; 213 (68.2%) participants fulfilled this requirement. Thus, 34.2% of the candidates successfully completed the experiment, making it moderately efficient. Data were collected for the other 99 participants who completed the experiment, but these were not included in the analyses. The rating scale had to be expanded for 50 (16%) and 30 (14%) of the 312 and 213 participants with and without successful completion of the practice trials, respectively. The following analyses are based on the data from the 183 participants without an expanded rating scale.

Two-way ANOVAs, with the algorithm and group (age × sex) as factors, were first applied to the three check variables: (1) the number of trials required in the training phase, (2) the number of trials that lasted longer than 60 s, and (3) the RMS error during the practice phase. None of the check variables showed any significant effect of the algorithm (1: *F*(2,189) = 0.08, *p* = 0.9255, *η*^2^ = 0.0007; 2: *F*(2,189) = 1.54, *p* = 0.2168, *η*^2^ = 0.0142; 3: *F*(2,189) = 3.23, *p* = 0.0419, *η*^2^ = 0.0285), the group, or the interaction between the algorithm and group. The results were the same if the analysis was applied to data from all 312 participants, who may not have completed the training phase as intended, instead of the 213 who successfully finished the training (1: *F*(2,288) = 1.01, *p* = 0.3654, *η*^2^ = 0.0066; 2: *F*(2,288) = 2.90, *p* = 0.0565, *η*^2^ = 0.01803; 3: *F*(2,288) = 1.17, *p* = 0.3133, *η*^2^ = 0.0073).

To examine whether the sensory system can evaluate information about viscosity from the local motion of light points, independent of static optical visual features, the actual ratings for the point-light movies (p-flow movies) given by each participant were fitted by linear regression to the expected ratings. Figure 2A shows the trial-wise ratings in the original, p-flow and LK movies. Figure 2B shows the participant-wise regression lines for the three movies. The slopes are mostly close to 1.0, indicating that the participants were able to extract information about the viscosity from the motion of point light displays.

**Figure 3:**
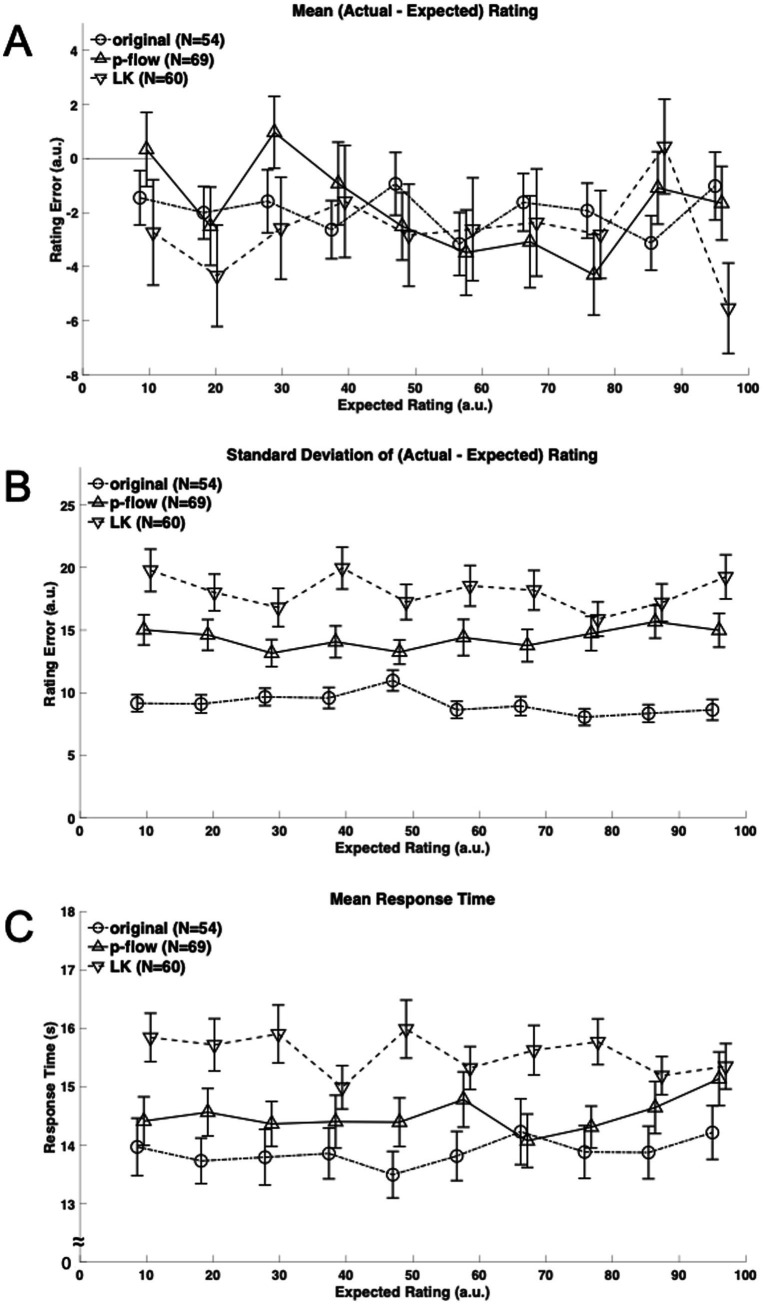
**(A)** Mean rating error defined as the difference between the actual and expected ratings is plotted against the expected viscosity rating ranks for each algorithm: the original, p-flow and LK movies with circles connected by broken lines, triangles by solid lines, and inverted triangles by broken lines, respectively. Note that there are shifts along the horizontal axis that were introduced between the algorithms merely for presentation purposes. **(B)** The standard deviation of the rating error across repeated presentations is plotted against the expected viscosity rating ranks for each algorithm: the original, p-flow and LK movies with circles connected by broken lines, triangles by solid lines, and inverted triangles by broken lines, respectively. **(C)** The mean response time across repeated presentations is plotted against the expected viscosity rating ranks for each algorithm: the original, p-flow and LK movies with circles connected by broken lines, triangles by solid lines, and inverted triangles by broken lines, respectively.

Figure 3A shows the mean rating error across repeated presentations for individual stimulus viscosities. The overall rating error was significantly less than, and therefore different from zero (*t*(539) = 5.597, *t*(689) = 3.950, and *t*(599) = 4.542, for the original, p-flow and LK, respectively; *p* < 0.0001 for all three cases) irrespective of the algorithm. The underestimation relative to the expected rating was larger with the least viscous-appearing stimuli (−12.46% and −15.40% for the least and the second least, respectively) but smaller with the other cases (between −5.38% and −1.37%). Nevertheless, ANOVA of the mean rating error did not show effects of the algorithm (*F*(2,180) = 0.2683, *p* = 0.7650, *η*^2^ = 0.001047), the stimulus viscosity (*F*(9,1620) = 1.087, *p* = 0.3690, *η*^2^ = 0.003835) or the interaction of algorithm and stimulus viscosity (*F*(18,1620) = 1.18, *p* = 0.2692, *η*^2^ = 0.008325). Thus, accuracy was not different between the algorithms.

Figure 3B, on the other hand, shows the standard deviation of the rating error across repeated presentation. ANOVA showed a significant effect of the algorithm (*F*(2,180) = 28.56, *p* < 0.0001, *η*^2^ = 0.1125), but the stimulus viscosity and the interaction of algorithm and stimulus viscosity were not significant (*F*(9,1620) = 0.8238, *p* = 0.5942, *η*^2^ = 0.002401; *F*(18,1620) = 1.007, *p* = 0.4488, *η*^2^ = 0.005868). *Post hoc* comparisons between the algorithms showed that all three differ from each other: the variability with repeated measurements was the smallest with the original, followed by the p-flow, and subsequently by the LK optical flow. Thus, precision or reliability was significantly better with the p-flow than with the LK optical flow.

Figure 3C shows the mean RT during the measurement phase. ANOVA showed a significant effect of the algorithm (*F*(2,180) = 5.630, *p* = 0.0042, *η*^2^ = 0.03801) but not the stimulus viscosity (*F*(9,1620) = 0.5731, *p* = 0.8201, *η*^2^ = 0.001109), or their interaction (*F*(18,1620) = 1.310, *p* = 0.1715, *η*^2^ = 0.005066). The RT was significantly longer for the LK optical flow movie than for the other two movies, suggesting difficulty in perceptual judgment when viewing the LK optical flow.

## 4 Discussion

The experimental results indicate that human observers can estimate viscosity from point light movies reconstructed by the p-flow algorithm as well as by the LK method with the same accuracy as from the original movie. This confirms that visual motion was an effective cue in viscosity perception (Kawabe et al., 2015), and that both algorithms provided such information. Whereas the source of the persistent error between the expected and the actual rating in all stimuli remains elusive, it may stem from an effect of the pre-processing applied to the original and the point-light display movies, namely, removed color and blurring by filtering on specific patterns of motion. Because it may warrant some reservation about the accuracy of the measurement, the issue must be addressed empirically in future studies. However, it is also evident that better precision, or more reliable perception was obtained with the p-flow than the optical flow extracted by the LK method, although the performance was the best with the original movie. In addition, whereas the estimation with the LK method was more difficult taking more time than the original movies with the optical features, the estimation with p-flow was not different from the original movie.

Note that the performance of the LK method may be better or worse depending on parameter tuning as well as the content of the movie, whereas the parameters were set in this study such that the number of moving dots, namely, motion energy as an approximation, was comparable between the algorithms. Whereas the LK method may lead to better results as more energy is contained in the movies, it is clearly indicated that the p-flow provides more efficient point-light display with a higher signal to noise ratio than the LK method.

Thus, the results show that the p-flow algorithm extracted and displayed visual motion of deforming fluid more effectively, in a framework that is compatible with both types of motion processing: feature tracking and gradient-based algorithms. The gap between the two types of motion becomes apparent in different contexts and can be described as follows: to extract motion, it is natural to use an algorithm that calculates the optical flow. For example, scene flow can be calculated theoretically under ideal conditions, and it has recently become possible to calculate optical flow accurately in the real world using deep learning tools such as RAFT (Teed and Deng, 2020). Biological motion stimuli are made by attaching light spots to human or animal joints and photographing them. Recently, various human movements have been accurately calculated with skeletal models using deep learning (Cao et al., 2021). Thus, in scene flow and biological motion, it is possible to calculate the motion of individual pixels and feature points, whereupon it is not difficult to create a point-light display. However, it is generally challenging to compute the motion of objects that do not have obvious landmarks, such as fluids. Whereas it might be possible to add beads, for example, to various fluids and to photograph them, this does not seem to be reproducible. Ideally, there would be some way to create computer graphics of a fluid with strictly defined parameters, from which psychologically plausible movements could be extracted.

Several methods of extracting optical flow have been proposed, including gradient- and feature-based approaches (Tu et al., 2019; differential techniques versus region-based matching as referred to by Barron et al., 1994; continuous optimization with prior versus discrete optimization, as corresponding to the categorization by Baker et al., 2011). While the gradient-based approach solves partial differential equations under the constant brightness assumption (Horn and Schunck, 1981; Lucas and Kanade, 1981), the feature-based approach links discriminative features in successive images over time (Wills and Belongie, 2004; Mac Aodha et al., 2013). The gradient-based approach is an ill-posed problem that cannot be solved unless a constraint encoding *a priori* information is incorporated in addition to the brightness constraint. The LK method, which is a gradient-based approach, calculates the optical flow by imposing the constraint that spatially neighboring regions move in the same manner. The spatial constraint that may be used in the gradient-based approach inherently introduces an *a priori* assumption about the spatial distribution, i.e., the shape of the object in motion, and therefore has limitations when applied to formless objects such as fluids. When no constraints on the spatial distribution are imposed, the motion at individual points or pixels should be spatially independent.

The p-flow algorithm is based on advection in fluid mechanics: the vector of the spatiotemporal derivative of an image translates between positions in successive frames consistently with the vector itself (Suzuki et al., 2017, 2020). Thus, one should find the same vector at the position implied by the vector itself in the following frame, if it comes from optical flow, not noise. The advantage of this formulation is that it can be applied at a point but not a finite-sized region of uniform velocity, and therefore, provides a description of the velocity distribution within a region that corresponds to the projection of a moving object. When a coherence or smoothness assumption successfully identifies the perimeter of the projection of a moving object in the scene, in contrast, the velocity distribution within the circumscribed region is lost because it is exactly what the assumption requires, although the velocity distribution is exactly what is needed to estimate how, or whether, the object in motion deforms at the same time. This is how conventional coherence (Lucas and Kanade, 1981) or smoothness constraints (Horn and Schunck, 1981; see Baker et al., 2011 for more prior and penalty terms for constraints), or co-segmentation approaches (e.g., Farneback, 2001) remain unsatisfactory.

The first to study non-rigid structure from motion was Ullman (1984). An incremental scheme was proposed in the study, where the initial data are used to generate an internal model and the model is gradually updated with subsequent data. Nonrigidity is introduced when the rigidity assumption fails. This study had a considerable influence on the field with others publishing on this subject (e.g., Terzopoulos et al., 1988; Torresani et al., 2008; Akhter et al., 2010; Pentland and Horowitz, 1991; Taylor et al., 2010). However, this scheme could not be maintained in its original form, not only because human perception of nonrigid structure was achieved without accumulation of view updates as the theory required (Braunstein et al., 1990), but also because accumulation of velocity information alone proved to be insufficient for a stable solution (Grzywacz and Hildreth, 1987). Whereas the last article may seem particularly relevant for the discussion here because it compares position- and velocity-based algorithms, the p-flow algorithm integrates position and velocity rather than choosing between the two. It needs to be examined in future studies what are critical differences between the incremental rigidity scheme, in which nonrigidity in image motion is primarily interpreted as the projection of rigid motion in the 3D space, and the p-flow algorithm, in which local and temporal rather than spatial rigidity is assumed.

There are a number of limitations in this study. This study only touched on the perception of viscosity and even then, p-flow was not perfect. P-flow should be tested on other tasks, like 3D structure from motion and compared to other algorithms in its actual performance as well as rigorous derivation in formulae in future studies. Although the participants were instructed to perform the experimental task in a dark room with the device held horizontally (landscape), there is no way of having rigorous control over the viewing conditions. Differences in screen brightness, ambient light, and visual acuity may have caused between-participant inconsistencies in stimulus perception. However, not only does this not directly explain differences between stimulus sets, but also there was a preliminary observation that suggests insensitivity, or tolerance of the perception, to these factors: when the same stimulus with visual noise is presented, the participants make the same response, although they report a more vivid impression with the stimulus without visual noise.

## 5 Conclusion

The p-flow over a large visual field is known to induce self-motion perception (Suzuki et al., 2019). In this study, the p-flow was presented on the small screen of a mobile device for viscosity perception. The experiment demonstrated that the p-flow provides cues for viscosity perception by human observers more effectively or efficiently than the conventional Lucas-Kanade method. Whereas the p-flow algorithm indicates how visual motion signals are integrated in time and space locally, further studies must be conducted using other types of stimuli and on other properties such as elasticity. While it is evident that some types of information that is efficiently extracted by the p-flow is effective in human visual perception of nonrigid motion of deforming fluid, and a mathematical definition of such information is provided, it needs to be empirically studied how general the finding is, and how such a specific type of information is detected and processed by the human visual system.

## Data Availability

The raw data supporting the conclusions of this article will be made available by the authors without undue reservation.
